# Emergence of the reproduction matrix in epidemic forecasting

**DOI:** 10.1098/rsif.2024.0124

**Published:** 2024-07-31

**Authors:** Hossein Gorji, Noé Stauffer, Ivan Lunati

**Affiliations:** ^1^ Laboratory for Computational Engineering, Empa, Dübendorf, Switzerland; ^2^ Chair of Computational Mathematics and Simulation Science, EPFL, Switzerland

**Keywords:** reproduction number, renewal equation, epidemic modelling

## Abstract

During the recent COVID-19 pandemic, the instantaneous reproduction number, *R*(*t*), has surged as a widely used measure to target public health interventions aiming at curbing the infection rate. In analogy with the basic reproduction number that arises from the linear stability analysis, *R*(*t*) is typically interpreted as a threshold parameter that separates exponential growth (*R*(*t*) > 1) from exponential decay (*R*(*t*) < 1). In real epidemics, however, the finite number of susceptibles, the stratification of the population (e.g. by age or vaccination state), and heterogeneous mixing lead to more complex epidemic courses. In the context of the multidimensional renewal equation, we generalize the scalar *R*(*t*) to a reproduction matrix, R(t), which details the epidemic state of the stratified population, and offers a concise epidemic forecasting scheme. First, the reproduction matrix is computed from the available incidence data (subject to some *a priori* assumptions), then it is projected into the future by a transfer functional to predict the epidemic course. We demonstrate that this simple scheme allows realistic and accurate epidemic trajectories both in synthetic test cases and with reported incidence data from the COVID-19 pandemic. Accounting for the full heterogeneity and nonlinearity of the infection process, the reproduction matrix improves the prediction of the infection peak. In contrast, the scalar reproduction number overestimates the possibility of sustaining the initial infection rate and leads to an overshoot in the incidence peak. Besides its simplicity, the devised forecasting scheme offers rich flexibility to be generalized to time-dependent mitigation measures, contact rate, infectivity and vaccine protection.

## 1. Introduction

The disruptive effects of the recent pandemic caused by the novel coronavirus SARS-CoV-2 have exposed our vulnerabilities regarding epidemic preparedness and response, reviving the scientific interest in mathematical modelling of the spread of disease [1–4]. Throughout the pandemic, the instantaneous reproduction number (or simply reproduction number, *R*(*t*)) has gained popularity and emerged as the primary measure of the state and evolution of the epidemic, upon which public health interventions are decided. Online monitoring systems were put in place to provide real-time estimate of the reproduction number [5,6], serving as the basis for public health decisions. Anticipating the epidemic growth by *R*(*t*) led to intervention measures that often had significant socio-economical impacts [7,8]. Therefore, the question of how closely the epidemic growth and *R*(*t*) are related has implications which may extend into broader societal and public health considerations.

Conventionally, the reproduction number is defined as the expected number of secondary cases that an individual would infect, if conditions remain unchanged [5,9]. It is a time-dependent property of the epidemic and is tightly related to the basic reproduction number, *R*_0_. The latter is the average number of secondary cases per primary infection when the disease is spreading in a fully susceptible population. It can be calculated as the largest eigenvalue of the reproduction matrix (or next-generation matrix) [5,10–13], and defines the epidemic threshold of the disease at given conditions: if *R*_0_ > 1 the number of infections grows exponentially, whereas the disease is not able to spread if *R*_0_ < 1.

It is appealing to extend this interpretation of *R*_0_ as a threshold parameter, which is a result from the linear stability analysis of the reproduction matrix, to the interpretation of *R*(*t*). Yet, real epidemics seldom follow or sustain an idealized exponential growth, less so over a long period of time [14]. An interpretation of *R*(*t*) analogous to that of *R*_0_ leads to an oversimplification, often far from real epidemic waves. The non-trivial interplay between the finite size of the population and the heterogeneity of the reproduction matrix raises questions about the relevance of such an analogy.

In the initial phase of the epidemic, the exponential growth arises due to the fact that the reproduction rate can be assumed to be constant. Even if interventions or behavioural changes are not imposed, this growth cannot be indefinitely sustained. Insofar as the number of infected individuals is relatively small, the decay in the number of susceptibles remains negligible. However, as the epidemic progresses, finite-size effects become important: the nonlinear relationship between the number of susceptibles and the previously infected individuals curbs the exponential growth and reduces *R*(*t*).

Finite size effects are anticipated in real epidemic waves and become more pronounced across heterogeneous populations, e.g. as a result of stratified contact patterns [15]. In the absence of preexisting immunity, for instance, the disease is initially driven towards individuals with a higher number of contacts (central nodes or hubs of the contact network). After their immunization, though, the propagation declines significantly, even if only a relatively small fraction of the total population has been infected. In other words, the initial exponential growth associated with the largest eigenvalue of the reproduction matrix persists only as long as the immunization of the corresponding compartments is negligible.

Following the linear stability analysis, the largest eigenvalue of the reproduction matrix is the most relevant to calculate *R*_0_ [11,12]. This is due to the fact that the corresponding eigenvector is expected to grow exponentially faster than the others in the asymptotic limit. Based on this result, it has been proposed to calculate *R*(*t*) from the largest eigenvalue of the reproduction matrix, at any given time *t* [16]. Although operationally well-defined, this interpretation of *R*(*t*) (which resembles a time-adaptive *R*_0_) may not provide an accurate understanding of the epidemic trajectory. In the case of finite-size heterogeneous systems, it is not guaranteed that the largest eigenvalue becomes dominant, as nonlinear effects might become significant sooner. Therefore, the entire reproduction matrix, along with the initial condition and preexisting immunity, has to be considered in order to characterize the epidemic evolution at a given time. In contrast to its prominent role as an epidemiological parameter, *R*(*t*) calculated from the dominant eigenvalue falls short of adequately describing the course of real epidemics. More importantly, it cannot be simply interpreted as an epidemic threshold, like *R*_0_, that determines a sustained exponential growth. Beyond the spectral radius, other compositions of epidemic statistics, e.g. consensus statistics in [17], have been pursued to devise more informative reproduction notions. While they remain useful and insightful for their targeted needs, e.g. to have a more reliable change point prediction in [17], these elaborated metrics still lack the integration with nonlinearity resulting from finite size effects, and therefore of limited use for epidemic projection where variation in susceptibility is pronounced. Such extensions focus on epidemic dynamic local in time and, at least in their current form, fall short in providing insight on the epidemic trajectory affected by nonlinearity.

Despite these difficulties, our main finding is that it is still possible to propose a simple measure of the generation of new infections which can then be used to quantitatively project the trajectory of epidemics taking into account both heterogeneity and non-linearity. Defining a multidimensional renewal equation that entails a heterogeneous infectiousness kernel, we devise a systematic strategy which offers an elaborated reproduction measure as well as its deployment for epidemic projections. First, we define a multidimensional renewal equation in which we make the dependency on the number of susceptible individuals explicit and introduce a heterogeneous infectiousness kernel which is a second rank tensor. Once it is estimated at a given time, the reproduction matrix can be used to forecast the epidemic dynamics at later times. To assess the effectiveness of this methodology, we apply the multidimensional renewal equation to predict synthetic and real-world epidemic waves, and we demonstrate that it offers a concise yet accurate predictive tool for epidemic forecasts.

## 2. Emergence of the reproduction matrix

### 2.1. Multidimensional renewal equation

In the absence of exogenous forces, the evolution of the epidemics at the calendar time, *t*, depends on the susceptibility of the population and on the number of infectious individuals, whose infectiousness varies with the time passed since infection, *τ* [9]. Owing to the inherent heterogeneity of the disease spread (e.g. due to stratification by age and the heterogeneity of the contact network) we derive a multidimensional renewal equation for the partitioned population. Let us denote by *I*_*i*_(*t*) the incidence time series (i.e. the number of new cases), by *S*_*i*_(*t*) the susceptibility, and by *N*_*i*_ the size of the population class, or cohort, *i* ∈ {1, …, *n*}. In the following, the subscript denotes the component corresponding to the *i*th class and we use bold notation for vector, and matrices, that describe the state of all classes. In general, the class may represent a group of individuals belonging to the same age group and characterized by the same vaccination state or other similar categories. We assume that the class to which an individual is assigned does not vary in time.

Assuming a Poisson process for the secondary infections, we write a generic renewal equation which governs the evolution of the incidence of the *i*th class,

2.1 $$I_{i}(t)=\sum_{\;j}\int_{0}^{\infty }\frac{S_{i}(t)}{N_{i}}\Theta_{ij}(t,\tau )I_{j}(t-\tau )\;d\tau ,$$

where Θ is the infectiousness kernel, which propagates the disease from infected individuals to the susceptible population and depends, in general, on the contact rate between individuals, disease transmissibility, and the duration of the infection. Notice that these quantities depend on the pathogen characteristics of the disease, on ambient parameters (e.g. ventilation [18–20]), and on the imposed protection measures (e.g. contact tracing, repetitive testing and administration of vaccines [21–24]).

Assuming that the time interval on which the incidence is defined is much smaller than the time required for reinfection, we can write

2.2 $${\dot{S}}_{i}(t)=-I_{i}(t),$$

and combine it with (2.1) to obtain

2.3 $$I_{i}(t)=\sum_{j}\int_{0}^{\infty }\Theta_{ij}(t,\tau )I_{j}(t-\tau )\;d\tau -\sum_{j}\int_{0}^{\infty }\int_{0}^{\infty }\frac{\Theta_{ij}(t,\tau )}{N_{i}}I_{i}(t-s)I_{j}(t-\tau )\;ds\;d\tau ,$$

where the first term on the right-hand side is a linear operator, and the second term is a quadratic operator that rectifies the growth and accounts for the finite susceptibility of the population. If the infectiousness kernel is independent of calendar time, the linear operator leads to an exponential growth of the incidence when the quadratic part is neglected. This is justified for infinitesimal relative incidences, *I*_*i*_/*N*_*i*_ ≪ 1, hence, whenever the incidence is negligible with respect to the size of the population, which is considered fully susceptible.

When nonlinear effects become important, however, a simplistic exponential projection of the epidemic wave is an overly pessimistic estimate of the actual course, as it fails to account for the moderating impact of the quadratic term. Note that the nonlinear term takes the form of a convolution which arises naturally when the distribution of the sum (here the cumulative sum of infected individuals) is considered (e.g. [25]).

### 2.2. Closure of the infectiousness kernel

With *i* ∈ {1, …, *n*}, equation (2.1) is a system of *n* equations. As the infectious kernel has more parameters than the number of equations, it cannot be estimated from the incidence time series without introducing a closure. As a first step, it is convenient to assume that the dependency of Θ on the calendar time and on the time passed since infection can be separated, as suggested in [9]. This implies that the pathogen-related infectivity is independent of *t* but depends on *τ*. Obviously, this is not necessary the case if, e.g. the infected individuals are isolated after symptom onset. Nevertheless, one could still split the course into different post-infection stages and apply the separability assumption to each sub-step (this is not pursued in this study).

We write the infectiousness kernel as the product of four contributions,

2.4 $$\Theta_{ij}(t,\tau )=f_{i}(t)C_{ij}(t)g_{j}\omega_{j}(\tau ),$$

where *ω*_*j*_ is the probability density of the generation-time distribution of the class *j* (which is normalized, i.e. $\int_{0}^{\infty }\omega_{j}(\tau )d\tau =1$); *g*_*j*_ = *β*_*j*_*τ*_*R*_ is the product between the average infectiousness *β*_*j*_ and its duration *τ*_*R*_; *C*_*ij*_ are the elements of the contact matrix, C [26], which describes the average number of people in the *i*th class that are in contact with a person in the *j*th class per unit of time (note that it fulfills the reciprocity condition *C*_*ij*_*N*_*j*_ = *C*_*ji*_*N*_*i*_ [27]); and *f*_*i*_ is a stratified prefactor that includes all the contributions which are not modelled by the other three terms (e.g. the impact of measures as well as seasonal forces). We observe that the effects of vaccine protection are described by a reduction of g (through β) along with a reduction of  f. The former contains the effect of the reduced infectivity of vaccinated individuals that have been nonetheless infected, whereas the latter account for the reduced infection risk of vaccinated individuals.

### 2.3. Reproduction matrix

The case reproduction number, *R*_*c*_(*t*), measures the average number of secondary infections generated by an individual infected at time *t*. *R*_*c*_(*t*) depends on the changes in individual behaviour and mitigation policy, and can be estimated only after the period of infection of the cohort [9]. A quantity more suited for real-time assessment of the epidemic is the instantaneous reproduction number, *R*(*t*), which measures the expected number of secondary infections generated by an individual infected at time *t*, if the circumstances remain unchanged [9]. In other words, *R*_*c*_(*t*) is a property of the individual infected at time *t*, whereas *R*(*t*) is the property of the epidemics at time *t* [9].

If applied to a population stratified into finite-size heterogeneous cohorts, the reproduction number derived for an homogeneous population will provide biased estimates. From (2.1), we can easily generalize the conventional instantaneous reproduction number for an heterogeneous and stratified population, and obtain the (instantaneous) reproduction matrix, R(t), of elements

2.5 $$R_{ij}(t)=\int_{0}^{\infty }\frac{S_{i}(t)}{N_{i}}\Theta_{ij}(t,\tau )\;d\tau =\frac{S_{i}(t)}{N_{i}}f_{i}(t)C_{ij}(t)\beta_{j}\tau_{R},$$

which represent the expected number of secondary infections in the *i*th class generated at time *t* by an individual in the *j*th class. Equation (2.5) takes into accounts the full heterogeneity of the system and explicitly describes the nonlinearity due to the reduction of the susceptible individuals in finite-size cohorts. Multiplying equation (2.5) by *I*_*i*_, dividing by the right-hand side of equation (2.1), and rearranging, we obtain

2.6 $$R_{ij}(t)=\frac{C_{ij}(t)\beta_{j}I_{i}(t)}{\sum_{k}C_{ik}(t)\beta_{k}\int_{0}^{\infty }\omega_{k}(\tau )I_{k}(t-\tau )\;d\tau },$$

which allows us to eliminate *f*_*i*_ (which describes contributions that are not explicitly modelled by the other terms in equation (2.4), such as the impact of mitigation measures, which are problematic to estimate *a priori*). If the contact matrix, the infectiousness vector, and the generation-time probability distribution are known, we can use equation (2.6) compute *R*_*ij*_(*t*) from the time series of the vectorial incidence.

The main extra model parameters introduced in addition to the homogeneous setting are the contact matrix and stratified infectiousness. Even though the contact matrix and infectiousness variability are assumed to be known throughout this study, those model parameters can be inferred from reported incidence data, under suitable assumptions. In practice, different schemes exist in order to estimate the model parameters, e.g. using annealing optimization combined with Bayesian posterior estimation [28]. We point out that the extra model coefficients fall into the linear identifiability framework, since the incidence at each time *t* depends linearly on the model coefficients. Treatment of the linear identifiability dates back to [29], and has been extended to the epidemic models (see, e.g. [30] for the case of linear compartmental models). We expect that under technical assumptions and provided incidence in *n* sub-groups, *n* model parameters can be identified, due to the linear structure of the model. However, rigorous treatment of this inference problem goes beyond the scope of the current study.

The reproduction matrix, R(t), allows us to compute the next generation of infectious individuals from the time series of the incidence vector, I(t). By substituting equation (2.4) into (2.1) we write

2.7 $$I_{i}(t)=\sum_{\;j}R_{ij}(t)\int_{0}^{\infty }\omega_{j}(\tau )I_{j}(t-\tau )\;d\tau$$

2.8 $$=\sum_{\;j}R_{ij}(t)\int_{0}^{\infty }\omega_{j}(\tau )\theta_{j}(t-\tau )I_{\mathrm{tot}}(t-\tau )\;d\tau ,$$

where we have introduced the fraction of incidence in the *j*-class with respect to the total incidence, *θ*_*j*_(*t*) = *I*_*j*_(*t*)/*I*_tot_(*t*), and summing over all cohorts we write the total incidence as

2.9 $$I_{\mathrm{tot}}(t)=\int_{0}^{\infty }\sum_{j}\sum_{i}R_{ij}(t)\omega_{j}(\tau )\theta_{j}(t-\tau )I_{\mathrm{tot}}(t-\tau )\;d\tau .$$

Note that the evolution of the epidemics does not depend only on R(t) and ω(t), but also on the distribution of the infection across the cohorts, θ(t), which varies over time. Therefore, it is not possible to characterize the epidemics simply by the largest eigenvalue of the matrix relating the vectors of the old and new generations of the infections (as done, for instance, in [16]), because the finite size of the cohorts may prevent the largest eigenvector from becoming dominant before nonlinear effects must be taken into account.

It is possible to derive a scalar reproduction measure, following the definition of the instantaneous reproduction number [9]. By comparing equation (2.9) and definition of the instantaneous reproduction number (see eqns (1) and (3) in [9]), it is straightforward to identify the scalar reproduction number

2.10 $$R_{s}(t)=\sum_{\;j}\sum_{i}R_{ij}(t)\int_{0}^{\infty }\omega_{j}(\tau )\theta_{j}(t-\tau )\;d\tau ,$$

which is simply the expected number of secondary infections caused by an individual infected at time *t*. If the incidence ratio varies slowly during the generation time (i.e. *ω*_*j*_(*τ*)*θ*_*j*_(*t* − *τ*) ≈ *ω*_*j*_(*τ*)*θ*_*j*_(*t*)), the scalar reproduction number simplifies to $R_{s}(t)=\sum_{j}\sum_{i}R_{ij}(t)\theta_{j}(t)$, which is equivalent to weighting each column of R(t) by the relative incidence of the corresponding cohort. In the general case of non-negligible generation time, however, it is not possible to substitute *R*_*s*_(*t*) in the renewal equation for the total incidence, (2.9), and the only meaningful approach is to use the full matrix R(t) to project the evolution of the epidemic into the future. In summary, different introduced notions of reproduction metrics are compiled in table 1. Note that while *R*_*s*_ conceptually offers a scalar reproduction number for heterogeneous settings, its evaluation and time evolution require the projection of R. Consequently, *R*_*s*_ may not be relevant for epidemic forecasting.

**Table 1.** Different notions of instantaneous reproduction numbers. The epidemic evolution in a heterogeneous population is controlled by R, which cannot be reduced, in general, to a scalar.

name	description	formula	relevance
reproduction matrix	the expected number of	equation (2.5)	accurate forecasting,
$R=\{R_{ij}\}$	secondary infections in *i*th class		given the contact matrix
	caused by an infected individual		and the infectiousness
	in *j*th class		
scalar reproduction	the expected number of secondary	equation (2.10)	unknown unless
number ***R***_*s*_	infections by an infected individual		R is given
	in a heterogeneous population		
homogeneous reproduction	the expected number of secondary	equation (4)	accurate forecasting,
number ***R***_*h*_	infections by an infected individual	in the electronic supplementary material	if a homogeneous
	in a homogeneous population		population is assumed

## 3. Results

The system of equations (2.1)–(2.4) serves as the basis to devise a scheme that allows forecasting of epidemic trajectories in heterogeneous populations. Note that the reproduction matrix introduced in the previous sections incorporates the entire information about the heterogeneity of the system, which dictates the course of the epidemic, and can be effectively embedded into the multidimensional renewal equation to accurately forecast the evolution of the disease in the population. In this sense, the reproduction matrix acts as a simple inference tool that brings together the data-driven estimates of the epidemic state (via (2.6)) and the mechanistic evolution of the incidence (via (2.1)). The integration of the two parts allows us to describe general epidemic trajectories that go well beyond the simplistic exponential growth which has relevance only in the linear regime of the epidemics. In comparison to recent studies, which addressed inhomogeneous epidemic models [16,31], our approach preserves both the heterogeneity and nonlinearity of the renewal equation.

Let us suppose that we have observed the course of the epidemic until the time *t*_0_, and that we would like to forecast the epidemic trajectory at a later time, *t* > *t*_0_. Let us also assume that *f*_*i*_(*t* > *t*_0_) = *f*_*i*_(*t*_0_) and *C*_*ij*_(*t* > *t*_0_) = *C*_*ij*_(*t*_0_) for all *i*, *j* ∈ {1, …, *n*}. Then, we have

3.1 $${\dot{R}}_{ij}(t)=\frac{{\dot{S}}_{i}(t)}{N_{i}}f_{i}(t_{0})C_{ij}(t_{0})\beta_{j}\tau_{R}=-\frac{I_{i}(t)}{S_{i}(t_{0})}R_{ij}(t_{0}),$$

which can be readily integrated to obtain the projection of the reproduction matrix

3.2 $$R_{ij}(t)=G_{t-t_{0}}[{\left\{I_{i}\right\}}_{\left[t_{0},t\right)}]R_{ij}(t_{0}),$$

via the transfer functional

3.3 $$G_{\Delta t}[{\left\{I_{i}\right\}}_{\left[a,a+\Delta t\right)}]=\exp\left(-\int_{a}^{a+\Delta t}\frac{I_{i}(s)ds}{S_{i}(a)-\int_{a}^{s}I_{i}(\tilde{s})\;d\tilde{s}}\right),$$

and a closed-form equation to forecast the incidence,

3.4 $$I_{i}(t)=G_{t-t_{0}}[{\left\{I_{i}\right\}}_{\left[t_{0},t\right)}]\sum_{j}R_{ij}(t_{0})\int_{0}^{\infty }\omega_{\;j}(\tau )I_{j}(t-\tau )\;d\tau .$$

The transfer functional G extrapolates the epidemic course to a given time *t* > *t*_0_ for which no observation are available, provided the initial reproduction *R*_*ij*_(*t*_0_) and the initial susceptibility *S*_*i*_(*t*_0_) that can be calculated from the incidence history *I*_*i*_(*t*) at time *t* ≤ *t*_0_ for all *i*, *j* ∈ {1, …, *n*}. As *R*_*ij*_(*t*_0_) is inferred through (2.6), the only unknown in (3.4) is *I*_*i*_(*t* > *t*_0_) for which the system can be iteratively solved. The merit of this approach is that, given the initial susceptibility and the incidence history prior to *t*_0_, the whole inference is encapsulated in the reproduction matrix $R(t_{0})$ and the future evolution is mechanistically modelled by a simple time-marching numerical discretization of the nonlinear integral (3.4) (see Material and methods and the electronic supplementary material for details of the solution method). We note that a simplified version of equation (2.1) has been considered in the age-specific renewal model of [32], based on the model proposed in [4]. In comparison, our forecasting approach given by equation (3.4), consistently combines the generic form of the reproduction matrix (2.6) and renewal equation (2.1), to integrate the epidemic statistics and forecasting. This is not the case for example in [4], where specific assumptions on the form of the reproduction number had to be introduced in order to close the renewal system. Moreover, our proposed approach goes beyond the contact pattern heterogeneity and includes variability in infectiousness, which is crucial for taking into account the effect of vaccination (due to different vaccination rates across various age groups).

To illustrate the importance of the reproduction matrix in describing the effects of nonlinearity and heterogeneity, and to assess the validity of employing it in the multidimensional renewal equation for epidemic forecast, we consider multiple test cases that encompass synthetic and real-world data. Our aim is to evaluate the predictive performance of the reproduction matrix for a population with age-stratified heterogeneity in the contact matrix and in the vaccination rate.

### 3.1. Synthetic epidemic waves

**Figure 1.**
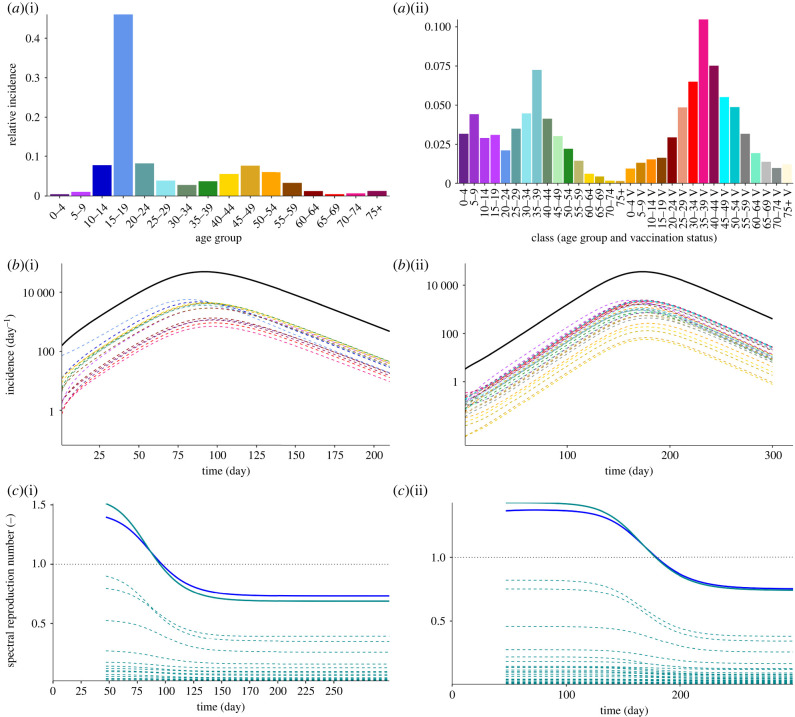
Synthetic case study of a fully susceptible (no vaccination) (1) and of a vaccinated (2) population. (*a*) Initial incidence of each class, defined according to age group (*a*)(i) and vaccination status (*a*)(ii), where ‘V’ refers to a vaccinated class. (*b*) Incidence profile as a function of time of the entire population (solid line) and of each class (dashed lines). Note that the same colour code is employed for the classes in sub-figures (*a*) and (*b*). (*c*) Temporal evolution of the eigenvalues (dashed lines) and spectral radius (solid green line) of the reproduction matrix, and homogeneous reproduction number computed (solid blue line).

**Figure 2.**
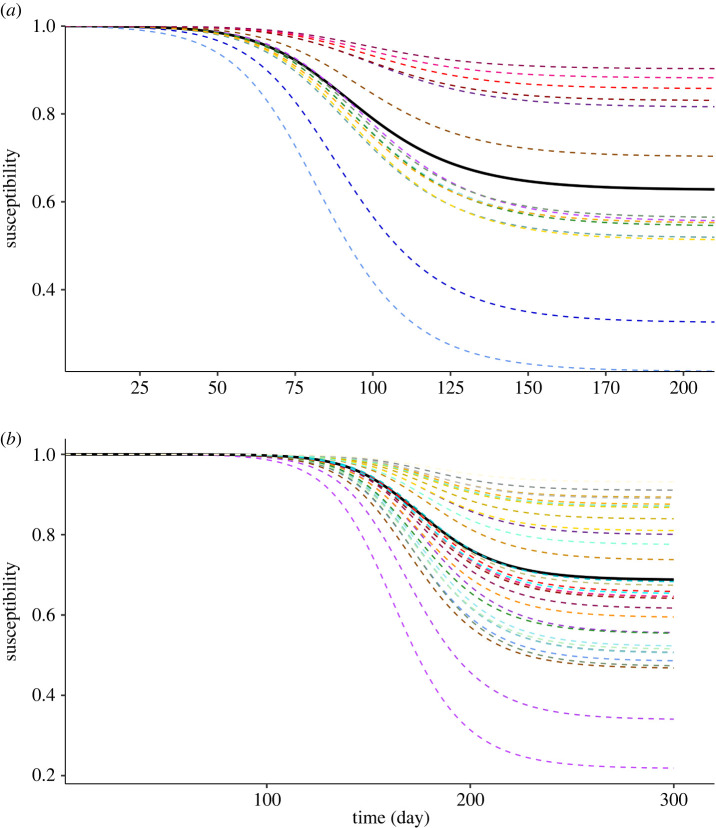
Decay in susceptibility in unvaccinated (*a*) and in vaccinated (*b*) population, per considered class (see figure 1*a*(i) and *b*(i) for the correspondence between age-group, vaccination status and employed colour code). Temporal evolution of the susceptibilities are shown for the entire population in solid line and of each class by dashed lines.

To emphasize the importance of the reproduction matrix to describe heterogeneous populations, we consider epidemic waves within a population that is consistent with the demography and contact patterns of Switzerland. We assume that mitigation measures remain unchanged during the wave and we exclude exogenous factors (see electronic supplementary material for the details about the input parameters of the model). We simulate two scenarios: a fully susceptible and a partially vaccinated population. In both cases, the population is initially exposed to an incidence that is stratified by age group (figure 1*a*). In the former scenario, the heterogeneity arises from the initial condition and the contact matrix, whereas in the latter, this heterogeneity is further intensified by stratified infectiousness, which results from varying vaccination rates. The epidemic courses are generated via an SIR (susceptible–infectious–recovered) compartmental model with parameters and next-generation matrix consistent with the heterogeneous renewal equation, (2.1). The simulated temporal evolution of the incidence of the total population and of each class are shown in figure 1*b* for the fully susceptible and vaccinated scenarios, respectively. Note that the epidemic trajectory results from the superposition of the evolution in the different cohorts, which reach the incidence peak at different times. As a result, different rates of reaching immunity per class is expected, as shown for the unvaccinated and vaccinated scenarios in figure 2*a*,*b*, respectively. For example in the unvaccinated scenario, shown in figure 2*a*, the decay in susceptibility of the age group 15–19 is the fastest, due to their largest contact rate and highest initial incidence.

From the full incidence history we can compute reproduction matrix, R(t), through (2.6) and calculate its eigenvalues, which are plotted in figure 1*c*(i) and *c*(ii), for the unvaccinated and vaccinated scenarios, respectively, and compared with the conventional reproduction number that is obtained by assuming a homogeneous population (see electronic supplementary material). As the reproduction matrix depends on time, the eigenvalues and eigenvectors also vary with time. In the scenario of a vaccinated population, two periods of time with almost constant eigenvalues are clearly visible (i.e. before day 100 and after day 250), as well as the corresponding exponential growth and decay of the incidence. In the fully-susceptible scenario, the exponential growth is less visible due to the early peak time, and the different cohorts exhibit different growth patterns. The spectral radius of the reproduction matrix has been proposed as a measure of the number of secondary cases for epidemics across heterogeneous populations instead of the conventional reproduction number that assumes a homogeneous population (e.g. [16]). We observe that these two quantities are relatively close, but the former is larger during the growth and smaller during the decay of the incidence in both scenarios. Besides, we notice that there exist eigenvalues with an order of magnitude similar to the spectral radius of R, without a counter part in the homogeneous version. As can be observed from right-hand side of equation (2.9), the full matrix R contributes to the total incidence and thus the contribution of R in the total incidence is not limited to its largest eigenvalue. The heterogeneous model offers us detailed insight into components of the reproduction matrix, *R*_*ij*_, as shown in electronic supplementary material. In the following, we demonstrate that this extra information carried by the full matrix is essential for faithful epidemic forecasting.

**Figure 3.**
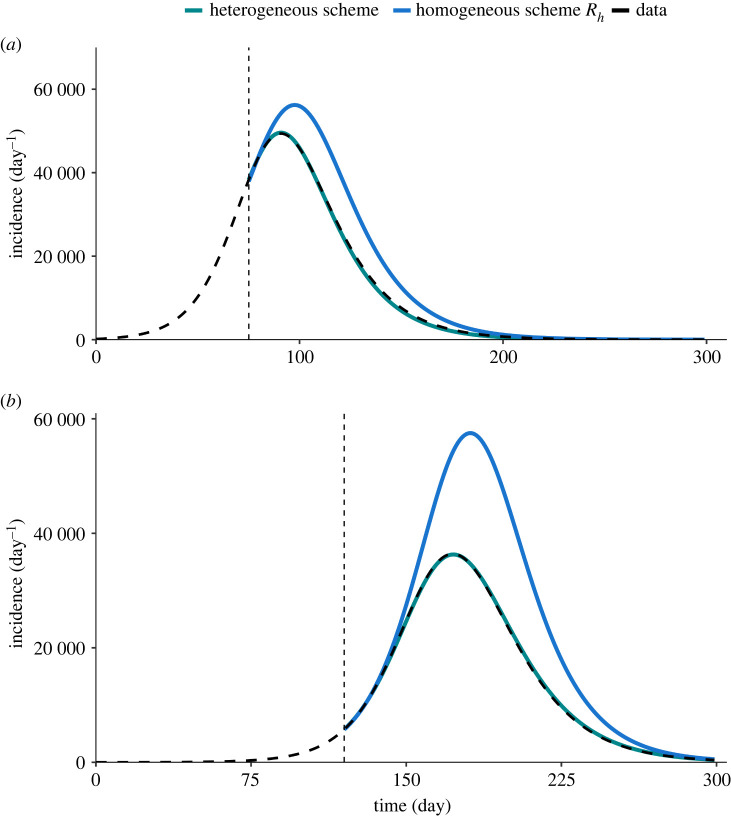
Forecast of synthetic epidemic course for the scenarios (*a*) without vaccination and (*b*) with vaccination. The forecast based on the use of the reproduction matrix in a multidimensional renewal equation (green line) and compared with the forecast based on the homogeneous assumption (blue line). The reference generated synthetic incidence is plotted by the thick dashed line, while the vertical thin dashed line indicates the time of the forecast.

**Figure 4.**
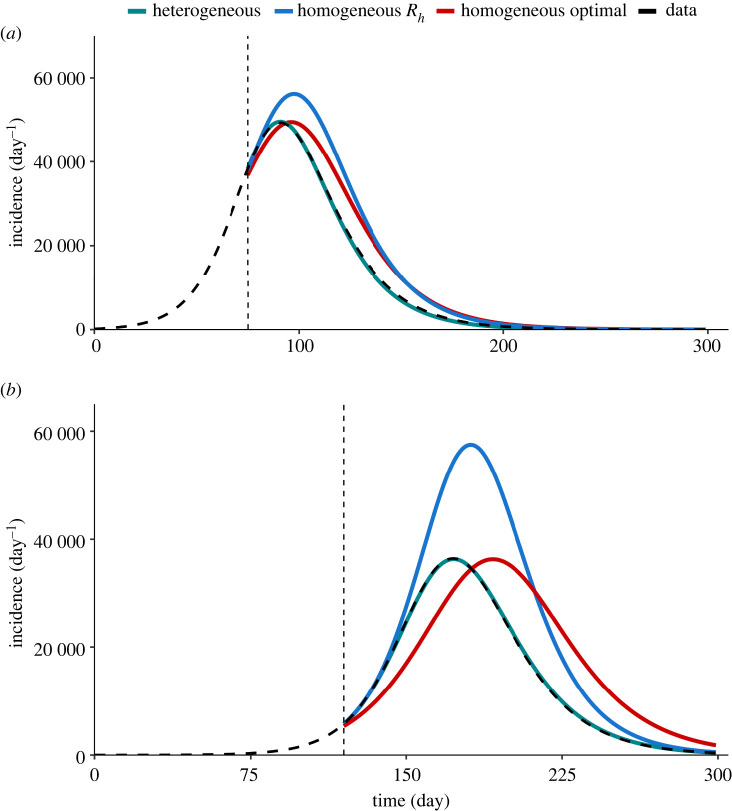
Forecast of synthetic epidemic course for the scenarios (*a*) without vaccination and (*b*) with vaccination. The forecast based on the use of the reproduction matrix in a multidimensional renewal equation (green line) is compared with the forecast based on the homogeneous assumption (blue), and with the optimized *R*_*h*_ (red). The reference generated synthetic incidence is plotted by the thick dashed line, while the vertical thin dashed line indicates the time of the forecast.

To demonstrate the importance of using the full information contained in R(t) to faithfully project the course of the epidemic, we compare the forecast based on the transfer functional (3.4), which takes into account the whole heterogeneity of the reproduction matrix, with the forecast based on the conventional reproduction number and the assumption of a homogeneous population (figure 3). In both scenarios, the forecast based on the full reproduction matrix is able to recover the course of the epidemic with high accuracy, proving the consistency of the projection scheme with the SIR model used for data generation. The homogeneous prediction, instead, significantly over-predicts the peak of the epidemic wave, as well as the total number of infections (which is proportional to the area below the curve). As the prediction is made in the growing phase, when the conventional reproduction number is smaller than the spectral radius, using the latter in the homogeneous renewal equation to forecast the epidemic trajectory would lead to an even higher estimate of the incidence peak. In order to better clarify the need for a heterogeneous description of the reproduction, we demonstrate in figure 4*a*,*b* optimal homogeneous projections where *R*_*h*_ is optimized as a free parameter to recover the correct peak incidence, for unvaccinated and vaccinated scenarios, respectively. Even for the optimal value (shown by red curves), the gap between the homogeneous and heterogeneous projections persists which further ensures the need to include heterogeneity for faithful forecasting. These simple synthetic scenarios show that a mode that is capable of describing the evolution of the different classes is necessary for an accurate forecast that correctly describes the heterogeneity and the nonlinearity of the epidemics, which becomes dominant in finite-size systems. In the discussed test scenarios, we examined recovery of epidemic curves where the imposed measures are assumed to be constant in time. In addition to these, we assess the model for a scenario involving more complex epidemic trajectory due to the variation of imposed measures. As demonstrated in the electronic supplementary material, the model can successfully replicate the complex course, if supplemented with additional data on exogenous factors.

### 3.2. COVID-19 pandemics: Omicron waves

While the synthetic scenarios exemplify well the limits of the homogeneous model and the importance of correctly accounting for the heterogeneity, application to real-world data still requires knowledge of parameters that are only partially known and remain uncertain in real epidemic waves. To validate the forecasting scheme with real data, we consider the recent COVID-19 waves in Switzerland and Scotland, caused by the Omicron variant at the beginning of 2022. Two key factors have been considered to choose these two datasets as benchmarks of real epidemic waves. Firstly, we would like to test the model in a situation in which there is minimal prior immunity within the populations. This enables us to reasonably estimate the susceptibility of the population by considering historical incidence data from a relatively short time frame. Therefore, we concentrate on the initial phase of the infection wave caused by Omicron, which was a variant characterized by the ability to escape the immune response of individuals that have recovered from previous variants. Secondly, since we do not incorporate the effects of mobility and spatial heterogeneity in the current forecasting scheme, we narrow our focus to relatively small spatial scales where time lags due to spatial epidemic waves can be neglected and the assumption of spatial homogeneity is reasonable. Accordingly, we concentrate our attention to two relatively small countries such as Switzerland and Scotland.

**Figure 5.**
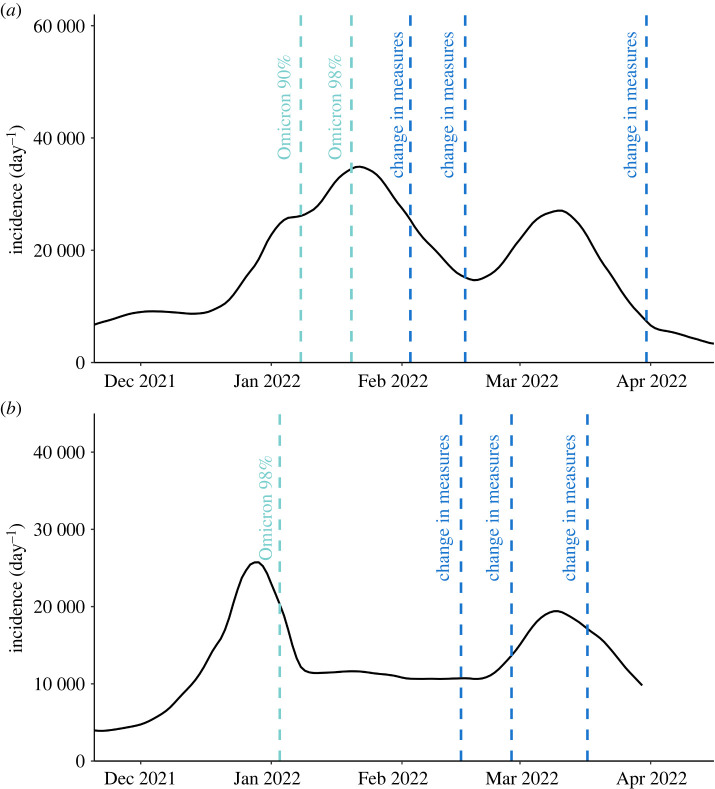
Epidemiological course of SARS-CoV-2 in (*a*) Switzerland [33] and (*b*) Scotland [34] during the period November 2021–April 2022.

The epidemiological background of both countries in terms of the imposed measures and emergence of the new variant is shown in figure 5. The initial onset of Omicron infections occurred in December 2021 and the variant has caused reporting of a large number of infections to April 2022. In order to exclude any concurrent impact of the Delta variant, we refine our analysis to consider solely the period when the Omicron variants became dominant (hence from January 2022 and February 2022 in Scotland and Switzerland, respectively). Moreover, we want to avoid periods that are characterized by significant changes in the imposed measures, which would require modelling the effect of time-dependent interventions. This limits our choice to the latter peak of infections that occurred in March–May 2022 in Switzerland, and March–April in Scotland.

**Figure 6.**
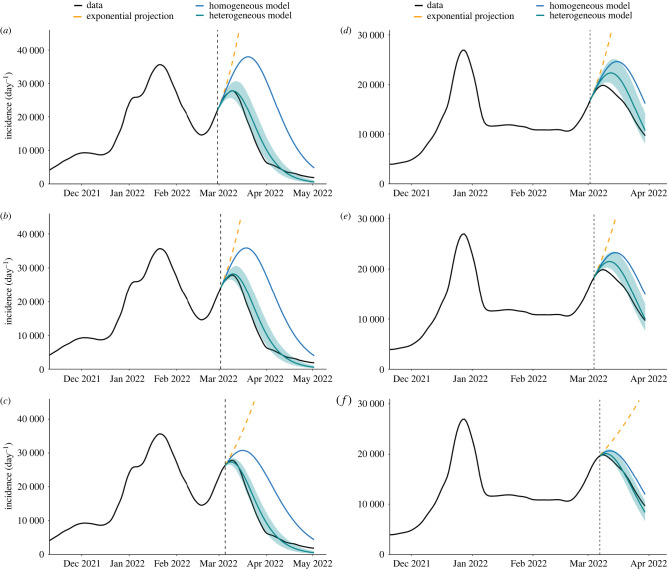
Forecast of the last Omicron waves in Spring 2022 during the COVID-19 pandemics in Switzerland (*a*–*c*) and Scotland (*d*–*f*). The plot compares the heterogeneous forecasting time-marching Monte Carlo scheme (green), homogeneous time-marching deterministic scheme (solid blue), and exponential extrapolation (dashed orange).

The epidemic forecasts for the selected Omicron incidence peaks in spring 2022 at three different starting dates are presented in figure 6. (The input parameters used in the forecasting scheme are listed in the electronic supplementary material.) We remark that only the incidence data prior to the forecast period are employed. To refine our simulations, the initial susceptibility is estimated from the asymptomatic fraction of infections (see the electronic supplementary material for details). To explore a wider set of parameters, we also conducted several sensitivity tests, which are reported in the electronic supplementary material, and confirm the robustness of the forecast results. We compare three types of forecast: a simplistic exponential extrapolation, a homogeneous forecasting scheme and the fully heterogeneous forecasting scheme, for which we also report a Monte Carlo evaluation of the 95% confidence interval (see Material and methods for the derivation of the Monte Carlo scheme and the electronic supplementary material for details of the algorithms). The increasing complexity incorporated by these three approaches allows us to understand the effect of non-linearity and heterogeneity both on the estimation of the reproduction matrix and on the forecast of the epidemic course.

A projection simply based on the scalar reproduction number is essentially an exponential extrapolation of the current epidemic state as it does not estimate the future evolution of the number of susceptible individuals. Obviously, this does not account for the finiteness of the population and is unable to provide any information about the height and the time of the epidemic peak. A more reasonable projection can be obtained based on a homogeneous renewal equation and the conventional reproduction number, as in the homogeneous forecast of the synthetic scenarios. This model accounts for the finite size of the system, but a clear overshoot in the prediction of the peak is observed in all tested scenarios. As the heterogeneity is neglected, the estimated reproduction number is attributed to the entire population, whereas it arises from contribution of the different cohorts that have different sizes and epidemiological behaviours. The most faithful projection is obtained with the heterogeneous forecasting scheme which is based on a multidimensional renewal equation and employs the full reproduction matrix. Note that this scheme provides an estimate of the uncertainty obtained by a Monte Carlo model ((5.5) and (5.6)). We observe that the estimated uncertainty, after the initial growth, shrinks once a significant decay in the susceptibility is attained. This is expected since the incidence fluctuations cannot exceed the number of susceptibles. As both the nonlinearity and heterogeneity are taken into account, we observe that the magnitude and the time of the peak become more realistic, particularly in the case of Switzerland, by incorporating the information from the full reproduction matrix. Finally, we remark that the model is not expected to remain accurate over a long time horizon, due to variations in model parameters and other exogenous factors [35]. This limitation can be alleviated, in principle, as such variations are supplied to the model with their corresponding mechanistic (or stochastic) rules.

## 4. Discussion

Intensified by the COVID-19 pandemic, numerous studies have addressed the problem of estimating the reproduction number during real-world epidemic waves and, concurrently, an extensive body of research has been dedicated to predicting the epidemic dynamics by means of tools that range from compartmental models to data-driven approaches (e.g. [28,36–40]). While exceptional progress has been made on both fronts, estimating the reproduction number and forecasting the epidemics have largely evolved separately. The widespread interpretation of the reproduction number calculated from incidence data remained that borrowed from the results of linear stability analysis (in analogy with *R*_0_). This interpretation assumes an underlying exponential growth, which is overly simplistic for capturing the dynamics of real epidemic waves in finite and heterogeneous populations. It is rather surprising that this interpretation persists, because the renewal equation, which serves to define *R*(*t*), is inherently an evolution equation capable of forecasting more complex and realistic scenarios.

Our study bridges this gap by defining a reproduction matrix R, which naturally extends the conventional reproduction number to heterogeneous populations and can be incorporated into a multidimensional renewal equation for epidemic forecasting. By explicitly describing the mutual effects of the classes in which the population is divided, R takes into account the full heterogeneity of the system that is neglected if the next-generation matrix is reduced to a scalar, either by assuming underlying homogeneity or considering solely its largest eigenvalue (note that this is justified only in the limit of an infinite population). Most important, the integration of R into a multidimensional renewal equation leads to a concise projection scheme. This enables us to predict realistic epidemic trajectories that consider the nonlinearity arising from the finite size of the cohorts, a crucial factor for accurate projections of the epidemic course.

We demonstrate the accuracy of the forecasting scheme in predicting the incidence profile both in synthetic test cases and from real datasets acquired during the COVID-19 pandemic. In the former case, the epidemic course can be recovered exactly. By contrast, if one uses a homogeneous model that does not employ the full reproduction matrix, the incidence peak is overestimated because the observed initial growth of the incidence is projected assuming that it can be sustained by the entire population. In reality, though, the reproduction number estimated from the incidence data is determined only by a fraction of the population (e.g. the most connected or the least protected) and the spread of the infection will slow down once they have been immunized. Note that employing the largest eigenvalue of the next-generation matrix will lead to an even higher incidence peak. The same trend is observed when forecasting real epidemic waves. As the model is subject to uncertainty in the input parameters, we quantify the uncertainty of the projected epidemic course based on a consistent stochastic treatment of all random contributions.

The heterogeneous and nonlinear renewal equation provides a compact description of epidemic courses that goes beyond simplistic exponential trajectories. The forecasting scheme takes into consideration the empirical data through the estimation of the reproduction matrix from the historical incidence and of the initial susceptibility. This requires independent estimates of the specific generation time, *ω*_*j*_, and the infectiousness, *β*_*j*_, of each class, as well as of the contact matrix, C (2.6). While the number of model parameters is enlarged compared to the standard homogeneous one, we observe that the model remains quite robust with respect to the extra uncertainty brought in by added input parameters. In particular, although the contact matrix is only partially known and its estimate is uncertain, the projected total incidence is rather insensitive to small variations of the contact matrix (e.g. to the differences between countries, as demonstrated in the electronic supplementary material). While the heterogeneous model has enough complexity to describe finite population groups that can sustain different infection rates, it is not possible to obtain a satisfactory description of the epidemic state, if the reproduction matrix is reduced to its largest eigenvalue (as done to calculate the conventional reproduction number), leading to a clear overshoot in the forecast peak incidence.

Even though we focus here on a stratification of the epidemic spread with respect to the age group and vaccination status, extension to different and more granular descriptions of the individual contacts can be incorporated into our framework. This may include, for instance, stratification into different geographical sub-regions, or accounting for additional mitigation measures that target only specific groups of individuals (e.g. to protect particularly the vulnerables). In particular, combination of our approach and the epidemicity index arising from spatial heterogeneity [41] is a relevant extension, in order to integrate the spatial fluxes into the proposed framework. We also envisage the possibility of devising schemes that try to learn the necessary model granularity from the data (hence, adding complexity only if necessary). Despite the complexity introduced by the additional model parameters, the linear dependency of the incidence on the heterogeneous model coefficients allows us to leverage linear model identification strategy and thus gives us opportunity to infer those model parameters for scenarios where they are only partially known. While not considered as the focus of this study, also time-dependent variations of imposed mitigation measures, contact rate, infectivity and vaccine protection can be included if reliable models can be derived from *a priori* knowledge or learned from historical data (see electronic supplementary material for time-varying measures). Additionally, the transfer of individuals across different classes (e.g. due to physical mobility) can be introduced.

The forecasting scheme is rich enough to allow future extensions and generalizations to include the above-mentioned aspects. In addition, our framework will facilitate the integration of compact epidemic models and incidence data into simple yet reliable prediction of the disease spread. The epidemic forecasting scheme can also be employed to expedite and simplify the inference of the unknown epidemic parameters. Finally, we also envision that our approach can be employed beyond epidemiology, to provide a novel framework for a rigorous combination of data and evolution operators for heterogeneous systems that can be described by renewal processes.

## 5. Material and methods

In addition to a given contact matrix, infectiousness profile, and initial susceptibility, three additional ingredients are needed to use (3.4) as a forecasting tool equipped with uncertainty quantification. First, we need to define a smooth representation of the incidence *I*_*i*_(*t*) of each cohort from their discrete daily counts $d_{i}^{\left(k\right)}$, where *k* ∈ {1, …, *n*_*d*_} indicates the number of the days with corresponding time stamps *t*^(*k*)^. Next, we need to devise the probability distribution of the estimated reproduction matrix $R(t_{0})$ with a sensitive uncertainty model. Finally, a stochastic process needs to be postulated to quantify the uncertainty of the forecasts.

### 5.1. Temporal filter

To prepare the case counts for the inference of the reproduction matrix and the successive projection of the incidence, we need to apply a temporal filter. While inferring the real-time incidence from observational data can be more accurately addressed in the Bayesian inference framework [42,43] or statistical methods [44], here we employ a simple approach based on temporal filtering, which is sufficient to demonstrate the importance of the reproduction matrix and the performance of the epidemic forecasting scheme for the test cases considered in this study. Admittedly, this approach might lead to biased estimates if used to process data close to an abrupt change in the reproduction number [6]. In this case, more advanced inference approaches should be used for the incidence data.

First, we map the discrete reporting onto a smooth version of the incidence,

5.1 $${\hat{I}}_{i}(t)=\sum_{k}K_{\theta }(t^{\left(k\right)},t)d_{i}^{\left(k\right)},$$

where $K_{\theta }()$ is a kernel parametrized by *θ*, which satisfies the normality condition. Next, the intermediate $\hat{I}$ is moved backward in time to account for the time lapse between the incidence and case identification. We assume that the time interval is composed of two parts: one is due to the infection to symptom onset, characterized by the incubation period; the other results from the time interval between symptom onset and medical confirmation. Let Π(s) be the probability density of having an elapsed time of *s* day from the infection to the case confirmation, then we get

5.2 $$I_{i}(t)=\int_{0}^{\infty }{\hat{I}}_{i}(t+s)\Pi (s)\;ds.$$

Obviously, the above operation introduces noticeable errors by backward spreading the incidence over a long time span if the delay to case confirmation is long and Π exhibits a long tail. In general, deconvolution methods need to be used to circumvent this issue, yet focusing on short delay to observation, deconvolution may not be necessary [6].

Once the kernel $K_{\theta }$ and the delay probability density Π are fixed, we get a smooth incidence time series suitable for the inference of the reproduction matrix and the forecast machinery.

### 5.2. Reproduction uncertainty

We follow Bayesian statistical inference to quantify the uncertainty of the reproduction matrix. To simplify the notations, let $R_{ij}=C_{ij}\beta_{j}{\hat{R}}_{i}$ where

5.3 $${\hat{R}}_{i}=\frac{I_{i}(t_{0})}{\sum_{\;j}C_{ij}\beta_{j}\int_{0}^{\infty }\omega_{j}(\tau )I_{j}(t_{0}-\tau )\;d\tau },$$

from which the reproduction matrix components can be readily recovered. Following [5] and assuming a Poisson process, the probability of the incidence *x* = *I*_*i*_(*t*_0_) conditional on the reproduction matrix and the historical incidence ${\left\{I\right\}}_{\left[0,t_{0}\right)}$ takes the form

5.4 $$\mathrm{Prob}[x|{\hat{R}}_{i},{\left\{I\right\}}_{\left[0,t_{0}\right)}]=\frac{{\left({\hat{R}}_{i}\Lambda_{i}\right)}^{x}}{x!}e^{-{\hat{R}}_{i}\Lambda_{i}},$$

where $\Lambda_{i}=\sum_{j}C_{ij}\beta_{j}\int_{0}^{\infty }\omega_{j}I_{j}(t_{0}-\tau )d\tau$. By postulating a gamma prior for ${\hat{R}}_{i}$ with parameters *a* and *b*, it is straightforward to show that the posterior distribution satisfies

5.5 $$\mathrm{Prob}[{\hat{R}}_{i}|x,{\left\{I\right\}}_{\left[0,t_{0}\right)}]\propto {\hat{R}}_{i}^{a+x-1}e^{-{\hat{R}}_{i}(b^{-1}+\Lambda_{i})}.$$

Therefore, various statistic model of $R_{ij}=C_{ij}\beta_{j}{\hat{R}}_{i}$ can be directly computed via samples generated from a gamma distribution with parameters $\left(a+I_{i}(t_{0}),{\left(b^{-1}+\Lambda_{i}\right)}^{-1}\right)$.

### 5.3. Uncertainty propagation

Given the case counts for the time interval 0 ≤ *t* < *t*_0_, we want to quantify the uncertainty of the epidemic trajectory that the model forecasts for times *t* ≥ *t*_0_. We postulate a stochastic dynamic which describes the randomness in the sense of a Poisson type process [45–48]. In particular, we assume that, at a given time, the number of infections generated by an infected individual is Poisson distributed. Let $I_{i}$ be the random variable denoting the incidence of the class *i* and $S_{i}$ its random susceptibility. For a given initial condition characterized by $R(t_{0})$, $I(t_{0})$, and $S(t_{0})$, we have

5.6 $$\begin{array}{cc} & I_{i}(t+dt)=\delta_{I_{i}}(t)\\ \mathrm{and}\; & dS_{i}=-I_{i}(t)\;dt, \end{array}\}$$

where the random process $\delta_{I_{i}}$, conditional on the history of the incidence ${\left\{I\right\}}_{\left[t_{0},t\right)}$, is Poisson-distributed with time-dependent expectation

5.7 $$E[\delta_{I_{i}}|{\left\{I\right\}}_{\left[t_{0},t\right)}]=G_{t-t_{0}}[{\left\{I_{i}\right\}}_{\left[t_{0},t\right)}]\sum_{j}R_{ij}(t_{0})\int_{0}^{\infty }\omega_{j}I_{j}(t-\tau )\;d\tau .$$

Starting from the initial conditions $I_{i}(t_{0})=I_{i}(t_{0})$, $S_{i}(t_{0})=S_{i}(t_{0})$ (which can be either deterministic or random), the devised continuous-time process is realized using a time-marching Monte Carlo scheme. Note that the process is non-Markovian with respect to the incidence because it depends on the historical incidence data.

Different statistical properties of the solution, including the variance, can be estimated directly from the generated samples. Furthermore, it is straightforward to incorporate into the Monte Carlo scheme the uncertainties on each components of the infectiousness *β*_*j*_, the generation time distribution *ω*_*j*_(*τ*), the reproduction matrix *R*_*ij*_(*t*_0_), as well as on the components of the incidence *I*_*i*_(*t*_0_) and susceptibility *S*_*i*_(*t*_0_). Upon postulating their priors, we run the process (5.6) where each random path employs an independent sample of the uncertain parameters.

### 5.4. Input parameters

The time-marching forecasting scheme consists of two elements:

1. The reproduction matrix $R(t_{0})$, which can be computed directly from the historical incidence ${\left\{I_{i}(t)\right\}}_{\left[0,t_{0}\right)}$, as the contacts *C*_*ij*_, the infectivity profile *ω*_*j*_(*τ*), and the average infectiousness *β*_*j*_ are known for all *i*, *j* ∈ {1, …, *n*}. In comparison to the conventional reproduction number estimates [5], here we need further data on the contact pattern as well as on the variation of infectiousness across the different population groups. As it is shown by a sensitivity analysis (see electronic supplementary material), knowing the exact specific form of the contact pattern is not crucial and an approximate understanding of the social mixing pattern is sufficient to obtain reliable estimates of *R*_*ij*_(*t*_0_) with an acceptable uncertainty. Note that the under-reporting of cases would have a negligible influence on the estimate of *R*_*ij*_ if its bias does not vary in time [49–51].
2. The transfer functional $G_{\Delta t}$ which projects into the future the state at the time of the forecast, characterized by the reproduction matrix $R(t_{0})$. To construct the transfer functional, we need to estimate the initial number of susceptibles. Although the under-reporting of cases does not directly impact the estimation of the reproduction matrix, it does affect the transfer functional. To mitigate this bias, we estimate the initial susceptibility by also taking into account the asymptomatic proportion of the infections (see electronic supplementary material). Note that under-reporting results in an overestimation of the initial susceptibility, which leads to forecasting a more severe epidemic wave than the real one, because the pathogen has more opportunities to circulate in the population. This intuitive picture is confirmed by several sensitivity tests presented in the electronic supplementary material.

### 5.5. Overview of numerical scheme

Finally, we describe the main steps of the deterministic forecast algorithm, whereas more details on stochastic treatment, homogeneous approximation and explicit modelling of vaccinated cases are provided in the electronic supplementary material. After computing the reproduction matrix from the filtered incidence (5.2) by means of (2.6), we employ a time-marching scheme to project the epidemic course into the future. The algorithmic steps are summarized in algorithm 1.

**Algorithm 1.** Heterogeneous time-marching forecast scheme.

**Input** $R_{ij}(t_{0})$, $I_{i}(t_{0})$, $\omega_{j}(\tau )$ and $S_{i}(t_{0})$, ∀i,j
Set $\tau_{c}$, $n_{\tau }$, and infection step size $\Delta t=\tau_{c}/n_{\tau }$
**for** $k=1\;:\;n_{t}$ **do**
$t_{k}\leftarrow t_{0}+k\Delta t$
Update the transfer functional, (3.3)
∀i: $G_{\Delta t}[{\left\{I_{i}\right\}}_{\left[t_{k-1},t_{k}\right)}]\leftarrow \exp\left(-\frac{I_{i}(t_{k-1})\Delta t}{S_{i}(t_{k-1})}\right)$

Update the reproduction matrix, (3.2)
∀i,j: $R_{ij}(t_{k})\leftarrow G_{\Delta t}[{\left\{I_{i}\right\}}_{\left[t_{k-1},t_{k}\right)}]R_{ij}(t_{k-1})$

Update the incidence, (3.4)
∀i: $I_{i}(t_{k})\leftarrow \sum_{j}R_{ij}(t_{k})\sum_{l=1}^{n_{\tau }}\omega_{j}\left(l\frac{\tau_{c}}{n_{\tau }}\right)I_{j}\left(t_{k}-l\frac{\tau_{c}}{n_{\tau }}\right)\Delta t$

Update the susceptibility,
∀i: $S_{i}(t_{k})\leftarrow S_{i}(t_{k-1})-I_{i}(t_{k})\Delta t$

Calculate the total incidence
$I_{\mathrm{tot}}(t_{k})\leftarrow \sum_{i}I_{i}(t_{k})$
**end for**

## Data Availability

Code are available from Zenodo: https://zenodo.org/records/11242746 [52]. The data are provided in the electronic supplementary material [53].
